# Supplementary material for: Enhancement of Resistive Switching Performance in Hafnium Oxide (HfO2) Devices via Sol-Gel Method Stacking Tri-Layer HfO2/Al-ZnO/HfO2 Structures
Source: Nanomaterials (Basel). 2022 Dec 22;13(1):39. doi: 10.3390/nano13010039 (PMC9823911; doi:10.3390/nano13010039)
Supplement: Supplementary file 1 [file nanomaterials-13-00039-s001.zip › nanomaterials-2042957-supplementary.pdf]

nanomaterials

MDPI

Supplementary Materials

Enhancement of Resistive Switching Performance in Hafnium Oxide (HfO_2) Devices via Sol-gel Method Stacking Tri-Layer HfO_2/Al-ZnO/HfO_2 Structures

Yuan-Dong Xu[1], Yan-Ping Jiang[1,*], Xin-Gui Tang[1], Qiu-Xiang Liu[1], Zhenhua Tang[1], Wen-Hua Li[1], Xiao-Bin Guo[1] and Yi-Chun Zhou[2,3,*]

[1] Guangzhou Higher Education Mega Centre, School of Physics and Optoelectronic Engineering, Guangdong University of Technology, Guangzhou 510006, China
[2] School of Advanced Materials and Nanotechnology, Xidian University, Xian 710126, China
[3] Frontier Research Center of Thin Films and Coatings for Device Applications, Academy of Advanced Interdisciplinary Research, Xidian University, Xi'an 710126, China
* Correspondence: ypjiang@gdut.edu.cn (Y.-P.J.), yichunzhou@xidian.edu.cn (Y.-C.Z.)

Figure S1 shows the pulse endurance of the Au/HfO_2/Al-ZnO/HfO_2/ITO device. After 120 pulse cycles, the high resistance state changes to the low resistance state and the resistive performance fails.

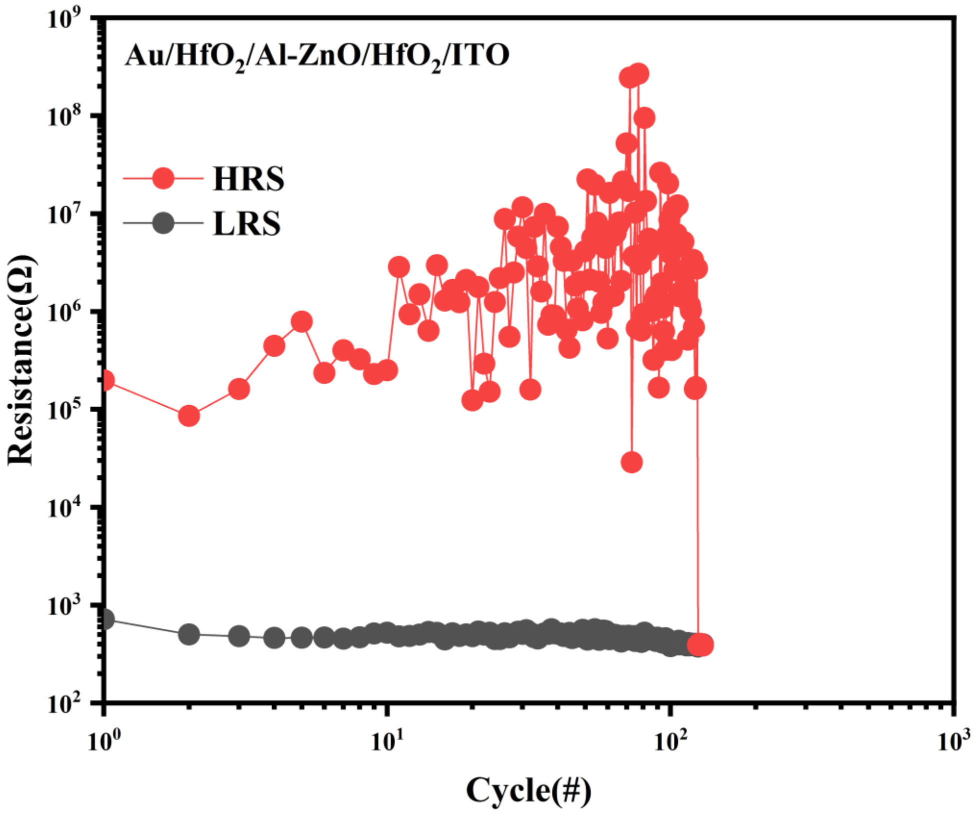

Figure S1. Pulse endurance of Au/HfO_2/Al-ZnO/HfO_2/ITO device.
